# The effects of music therapy on intraoperative and postoperative parameters: A randomized single-blind study

**DOI:** 10.1097/MD.0000000000043840

**Published:** 2025-08-22

**Authors:** Murat Bayram Kaya, Arzu Esen Tekeli, Nurettin Kurt, Mehmet Emin Keskin, Ali Kendal Oğuz

**Affiliations:** a Department of Anesthesiology and Reanimation, Van Training and Research Hospital, Van, Turkey; b Department of Anesthesiology and Reanimation, Van Yuzuncu Yil University, School of Medicine, Van, Turkey; c Department of Anesthesiology and Reanimation, Health Science University, Gazi Yaşargil Training and Research Hospital, Diyarbakir, Turkey.

**Keywords:** BIS values, general anesthesia, music therapy, thyroidectomy, VAS scores

## Abstract

**Background:**

Music therapy has been used in medicine to reduce patient stress and to improve mood. This study aimed to evaluate the effects of music therapy on intraoperative hemodynamics and medication requirement and postoperative pain and side effects.

**Methods:**

Eighty patients with American Society of Anesthesiologists I to II physical status at the ages of 20 to 60 for whom elective thyroidectomy surgery was planned were included in the study. General anesthesia was induced for patients and demographic data were recorded. The patients were randomly divided into 2 groups. The groups were determined as music group (group M) and control group (group C). The intraoperative vital signs of the patients (heart rate, blood pressure, and oxygen saturation), bispectral index values, train-of-four neuromuscular monitoring values, additional opioid and muscle relaxant requirements, and complications were recorded.

At the end of the operation, extubation was performed following standard decurarization using atropine and neostigmine. The 0th hour, 3rd hour and 6th hour visual analogue scale scores of the patients were measured and recorded.

**Results:**

Intraoperative fentanyl and rocuronium consumption were found to be approximately 23% lower in group M compared to group C (*P* < .05).The bispectral index values of the patients were similar between the groups (*P* > .05). It was also observed that the postoperative pain levels of the group M were lower (*P* < .05). Music therapy was determined to not create a difference in terms of the blood pressure, heart rate, and saturation (SpO_2_) levels during recovery from anesthesia (*P* > .05)

**Conclusions:**

Playing music, which is a non-pharmacological intervention, is an effective method without a side effect that not only reduces the intraoperative need for muscle relaxant and analgesic use but also causes positive effects on postoperative visual analogue scale scores.

## 1. Introduction

**Figure 1.**
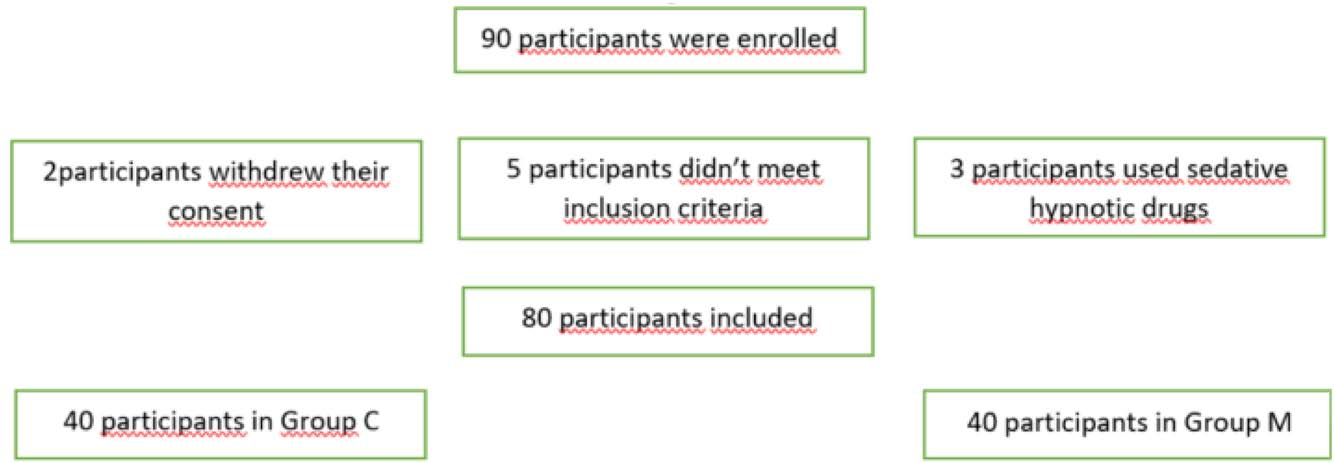
Flow diagram illustrating the random allocation of patients into 2 equal groups: the music group (M), who received intraoperative music therapy through headphones, and the control group (C), who wore identical headphones with no audio content. The diagram reflects patient recruitment, randomization, exclusions, and final inclusion in statistical analysis according to CONSORT guidelines.

Research has consistently shown that anxiety among surgical patients can lead to heightened concerns regarding recovery and pain management, especially during anesthesia.^[1]^ These concerns can elevate stress levels, which may result in fluctuations in hemodynamic parameters. Moreover, the intensity of postoperative pain significantly impacts the quality of life during early recovery.^[2]^ Although pharmacological agents are commonly used to manage anxiety and pain perioperatively, these medications may have substantial side effects, limiting their use. Consequently, non-pharmacological alternatives, such as music, have emerged as promising options due to their cost-effectiveness and minimal side effects. Studies have demonstrated that perioperative music interventions can positively influence pain degree after surgery, anxiety, usage of sedatives during surgery, and opioid consumption.^[3,4]^ Music played during general anesthesia has been shown to significantly reduce postoperative pain and opioid consumption in the early postoperative period.^[5]^ Furthermore, music can help mitigate the physiological stress response to surgery, evidenced by decreased cortisol and cytokine levels in patients undergoing locoregional anesthesia combined with sedation.^[6]^ Despite these benefits, research on the clinical outcomes of music interventions remains limited. Music therapy, as a simple and easily implementable option, has the potential to enhance patient satisfaction and could be integrated into multimodal analgesia strategies^[7]^ (Fig. 1).

While numerous studies have supported the role of music in reducing anxiety, sedative requirements, and postoperative pain,^[3–6]^ other research has raised questions regarding the influence of passive listening or the role of auditory stimuli independent of music itself. Given this ambiguity, classical instrumental music—known for its calming and structured acoustic properties—is hypothesized to reduce anesthetic requirements and enhance recovery.

The role of music therapy has been linked to improved hemodynamic stability and a reduction in postoperative complications, particularly in abdominal and cancer surgeries. Additionally, music can facilitate extubation in intensive care units and promote relaxation during spinal anesthesia.^[8,9]^ However, some researchers question its efficacy, pointing to factors such as passive listening experiences and the role of staff in selecting music. Moreover, it remains unclear whether the benefits of music therapy are solely due to music or if auditory stimuli at certain frequencies could yield similar effects.^[10]^

Given the conflicting evidence surrounding the impact of music during surgeries performed under general anesthesia, we hypothesize that classical music may have a calming effect, potentially reducing the need for anesthetics during surgery. This study aims to investigate how music influences to depth of anesthesia, non invasive blood pressure, heart rate, SpO_2_ values during surgery, consumption of muscle relaxant and analgesic, pain levels, nausea, vomiting, and tremors during early postoperative recovery for patients will be operated due to thyroidectomy under general anesthesia. Both train-of-four (TOF) and bispectral index (BIS) monitoring were utilized to evaluate the impact of music on muscle relaxant usage and anesthesia depth.

## 2. Patients and methods

### 2.1. Study design

This two-arm, randomized, controlled, single-blind study implemented at Van Yüzüncü Yil University Faculty of Medicine. The study was approved by the Van Yüzüncü Yil University Non-Interventional Clinical Research Ethics Committee (2021/08-06) and registered under Clinical Trials number NCT05886231, following CONSORT guidelines.

### 2.2. Participants

Eligible patients were informed about the study preoperatively by the anesthesiologist. Adults aged 20 to 60 years, classified as American Society of Anesthesiologists I–II, and scheduled for elective thyroidectomy were included the study. Exclusion criteria included patients with hearing impairments, those using hearing aids, individuals unwilling to listen to unfamiliar music, emergency conditions or bleeding, cognitive disorders (e.g., dementia, intellectual disabilities), and obesity (body mass index ≥ 30). Additionally, patients on systemic steroids, immunosuppressive drugs, or cytotoxic medications were excluded, as these could influence the physiologic answer to surgery.^[11]^ Only medications related to chronic conditions, such as diabetes or hypertension, were administered on the day of surgery.

A team of academician from Van Yüzüncü Yil University conservatory department created a 3-hour playlist of classical music. If the surgery exceeded 3 hours, the playlist automatically repeated. The control group used the same noise-canceling headphones as the intervention group but a CD without music was played throughout the procedure.

After the induction of general anesthesia and before the skin incision, active noise-canceling Sony WH-CH510 Bluetooth headphones were placed on all patients and connected to an MP3 player. The music or silence, depending on group assignment, was played continuously until wound closure. The music or silence volume was standardized at 85 dB, aligning with the upper safety threshold recommended by the World Health Organization to prevent hearing damage in occupational settings (WHO, 1997). Although some perioperative studies advocate for lower volumes such as 60 to 70 dB to enhance comfort,^[7]^ 85 dB was selected to ensure audibility over ambient noise and consistent delivery through noise-canceling headphones.

Intraoperative consumption of opioids, sedatives, and neuromuscular blockers was monitored from the moment the headphones were applied until they were removed.

General anesthesia was induced with propofol (2–3 mg/kg), fentanyl (1 μg/kg), and rocuronium bromide (0.6 mg/kg). Patients were monitored using BIS and TOF, and anesthesia was maintained with desflurane (4%–6% end-tidal) in a 40% O_2_ and 60% air mixture. The volume of music or silence was set to 85 dB to prevent hearing damage.

### 2.3. Perioperative care

Upon entering the operating room, standard monitoring (noninvasive blood pressure, ECG, and pulse oximetry) was initiated. BIS and TOF were used to assess anesthesia depth and neuromuscular blockade. General anesthesia was induced with propofol (2–3 mg/kg), fentanyl (1 μg/kg), and rocuronium bromide (0.6 mg/kg), and maintained with desflurane (4%–6% end-tidal) in a 40% O_2_ and 60% air mixture. Tramadol (1 mg/kg) and paracetamol (20 mg/kg) were administered for postoperative analgesia. Extubation was performed using atropine and neostigmine.

### 2.4. Outcomes

The impact of music on opioid and muscle relaxant consumption, as well as hemodynamic parameters was the main outcome. Pain assessment with visual analog scale at 0, 3, and 6 hours postoperatively and side effects such as nausea, vomiting, chills, itching, and desaturation were evaluated as a secondary outcome.

### 2.5. Randomization

Eligible patients were informed and randomized by an independent anesthesiologist using computer-generated randomization tables and sealed opaque envelopes into music (group M) or control (group C) groups. The study followed a single-blind protocol: patients were unaware of group allocation, and outcome assessors were blinded. One day before surgery, patients underwent preoperative evaluation, provided consent, and routine tests were completed.

Subsequently, after anesthesia induction and before skin incision, active noise-canceling headphones were placed on all patients. The intervention consisted of either classical music or silence, depending on group allocation.

Patients who met the inclusion criteria and signed the consent form were divided into music (group M) and control (group C) groups by a computer using a randomization table by an anesthesiologist who was not involved in the study. A total of 80 patients participated, with 40 patients in each group (Fig. 1). Randomization was performed using closed envelopes to assign patients to their respective groups. Three patients were excluded due to the use of hypnotics (benzodiazepines), 2 patients were excluded due to dissatisfaction. According to the power analysis, 40 participants were planned for each group (Fig. 1). Envelopes for assignment to the groups were randomized by the patient. One day before surgery, preoperative evaluation and necessary examinations were performed in the anesthesia outpatient clinic. Informing about the study and obtaining a consent form were completed. No request other than routine tests was made before surgery.

### 2.6. Sample size calculation

When determining the sample size, a two-point scale with an error rate of (α) 0.05 type 1 and a power of 80% to detect the difference between 2 groups was used. According to this calculation, the sample size for each group was determined as 40.

### 2.7. Statistical analysis

Descriptive analysis including mean, standard deviation, median, and ratio values was performed for both groups and the results were recorded. Mann–Whitney *U* test was used for quantitative data, chi-square test was used to evaluate qualitative data and Kolmogorov–Smirnov test was used for distribution of variables. Statistical significance was taken as *P* < .05 and SPSS 27.0 program was used.

## 3. Results

The study included 80 patients, with equal distribution into the music (group M) and control (group C) groups. Since the population had a normal statistical distribution, independent *t* tests were used to compare mean age and body mass index, and no significant differences were found between the 2 groups. Chi-square tests showed no significant difference in terms of sex distribution, with equal numbers of males and females in both groups. Demographic data, including age, gender distribution, height, weight, body mass index, American Society of Anesthesiologists score, heart rate, systolic arterial blood pressure, diastolic arterial blood pressure, SPO_2_, BIS, and TOF values, did not differ significantly between the groups (*P* > .05) (Table 1).

**Table 1:** Demographic data.

			Min–Max	Median	Mean ± SD/n (%)	
Age			21.0–60.0	45.0	45.6 ± 11.0	
Gender		Female			72	90.0%
		Male			8	10.0%
Height			150.0–186.0	160.0	161.5 ± 8.1	
Weight			50.0–105.0	78.0	74.8 ± 10.3	
BMI			20.8–43.1	29.3	28.7 ± 3.3	
ASA	I				9	11.3%
	II				71	88.8%

Mann–Whitney *U* test was used.

ASA = American Society of Anesthesiologists, BMI = body mass index.

**Figure 2.**
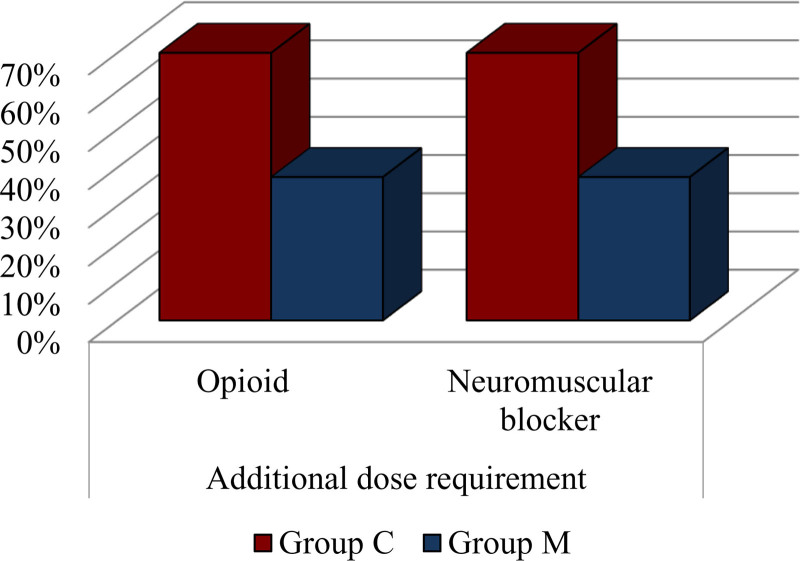
Comparison of the number of patients requiring additional intraoperative opioid and neuromuscular blocker doses between the music group (M) and the control group (C). The figure illustrates a statistically significant reduction in additional dose requirements in the music group, suggesting a potential intraoperative sparing effect of music therapy.

Figure 2 indicates that the need for additional opioid and muscle relaxant doses was significantly lower in the music group compared to the control group (*P* < .05). There was no significant difference between the groups in terms of postoperative analgesic need and undesirable effects (*P* > .05) (Table 2 and Fig. 2).

**Table 2:** Additional dose requirements and side effect status.

		Group C		Group M		*P*-value	
		n	%	n	%		
Additional dose requirement							
Opioid		28	70.0%	15	37.5%	**.004**	*X*²
Neuromuscular blocker		28	70.0%	15	37.5%	**.004**	*X*²
Postoperative analgesia requirement	(−)	0	0.0%	0	0.0%	1.000	*X*²
	(+)	40	100.0%	40	100.0%		
Side effects	(−)	40	100.0%	40	100.0%	1.000	*X*²
	(+)	0	0.0%	0	0.0%		

Bold values indicate statistical significance. Mann–Whitney *U* test was used. *X*² = chi-square test.

**Figure 3.**
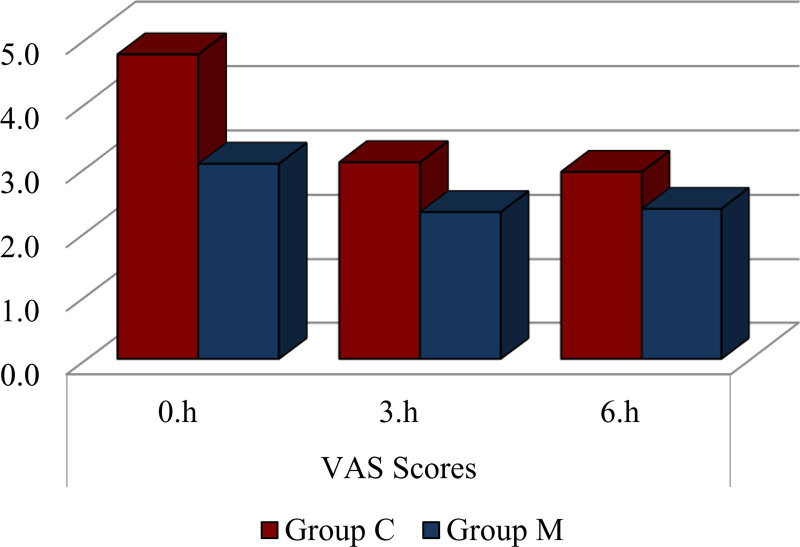
Visual analog scale (VAS) pain scores recorded at 0, 3, and 6 hours postoperatively in the music group (M) and the control group (C). The figure demonstrates consistently lower pain scores in the music group at all time points, indicating the analgesic benefits of intraoperative music therapy in the early postoperative period.

Postoperative pain intensity, assessed using the visual analogue scale method, demonstrated significantly lower pain scores in the music group compared to the control group (*P* = .000). Specifically, 0th-hour, 3rd-hour, and 6th-hour visual analogue scale scores in the M group were significantly lower than those in the control group (*P* < .05) (Fig. 3).

These findings indicate that intraoperative music significantly reduced the need for opioids and muscle relaxants, contributing to lower postoperative pain levels.

The duration of anesthesia was longer in the music group; however, the difference between the 2 groups was not statistically significant.

## 4. Discussion

Intraoperative and postoperative evaluations of thyroidectomy patients who were exposed to instrumental classical music were compared with patients who were not exposed to music but were protected against ambient sound. The simplicity and ease of implementation of intraoperative music make it an appealing option for improving patient experience and outcomes. Our findings demonstrate statistically significant reductions in postoperative pain and a decreased need for intraoperative opioid and neuromuscular blocker medications. However, there was no substantial difference in postoperative analgesic use or adverse effects.

Research on the benefits of music in reducing perioperative anxiety, anesthetic or sedative use, and pain relief has yielded inconsistent results. A comprehensive review by Matsota et al highlighted that the effectiveness of music largely depends on individual patient characteristics.^[12]^ Prior studies have assessed the impact of music on anxiety before and after surgery. For example, Ajorpaz et al (2011) reported that playing music in the waiting room prior to surgery reduced anxiety, while Koch et al found similar benefits during spinal anesthesia.^[13,14]^

Consistent with our results, Choi et al’s crossover clinical trial demonstrated significant pain reduction for patients undergoing cataract surgery when exposed to music.^[15]^ Additionally, Koch et al observed decreased pain levels among patients undergoing lithotripsy when exposed to intraoperative music alongside patient-controlled intravenous opioid analgesia.^[14]^ These findings align with previous research suggesting that music can effectively reduce pain and opioid consumption during surgery.

Conversely, Szmuk’s study found that the required sevoflurane concentration to maintain a BIS score near 50 during laparoscopic cholecystectomy remained unchanged regardless of music exposure.^[16]^ Fewer anesthetic agents can reduce the risk of hemodynamic fluctuations, leading to smoother recovery from anesthesia. While several studies have shown positive effects of music on postoperative pain and opioid use, our results are consistent with studies that showed lower postoperative pain and reduced opioid requirements in patients exposed to music during surgery.^[17,18]^

It could be argued that using readily available playlists based on genre might be sufficient. However, within any genre, there is a vast diversity of musical pieces, some of which may not align with the patient’s personal preferences. In prior studies investigating the effects of intraoperative music, researchers selected the music in advance.^[17,19,20]^

No studies have specifically used patients’ preferred music playlists during surgery, though 2 studies allowed patients to choose from a limited selection of prechosen tracks.^[7,17,21]^ However, it is unclear which specific music the patients selected or preferred in these studies. An evaluation of 85 randomized controlled trials demonstrated positive effects of instrumental music. Few studies have allowed patients to choose their music. Two studies permitted limited patient selection from a preselected list of tracks, but it remains unclear which specific music was chosen.^[22]^ To ensure standardization, we have chosen a list of soft, instrumental music. Noise, a universal stressor in surgical settings, was controlled through the use of noise-canceling headphones in both groups. This may have influenced the observed effects of music, as reducing noise alone could diminish the stress-relieving benefits attributed to music.^[17,20]^ Although noise-canceling headphones were used in both groups to control ambient noise, this standardization ensured that observed differences could be attributed to music rather than noise reduction alone. Previous studies suggest that even after controlling for environmental noise, music retains intrinsic anxiolytic and analgesic properties.^[23]^

Few studies have examined the impact of music on postoperative complications. In line with prior research, our study found no significant differences in complication rates between the music and control groups. Additionally, preoperative administration of benzodiazepines was excluded, minimizing the influence of premedication on auditory stimuli perception.

The fact that the perioperative team was unaware of the groups, group assignments were made by an anesthesiologist who was unaware of the study and the statistician was blinded to the study are the strengths of our study. These measures reduced potential bias, ensuring that outcomes were evaluated by healthcare professionals blind to the intervention. Furthermore, the use of multiple pain assessments at 0, 3, and 6 hours post-surgery increased the likelihood of detecting significant differences.

However, limitations include a relatively small sample size, which raises the risk of false positive findings, and variations in individual responses to music. Future studies should focus on larger sample sizes and specific types of surgery to further examine music’s effects. Additionally, research into the impact of patient music preferences is warranted to assess whether personalized playlists yield different results under general anesthesia.

## 5. Conclusion

In summary, our study highlights the potential benefits of intraoperative music in reducing the need for opioids and neuromuscular blockers while improving pain outcomes following thyroidectomy. Despite its ease of implementation, low cost, and safety as a non-pharmacological intervention, the study suggests that its impact on surgical and recovery outcomes may be limited. Future research should continue to explore the influence of music on patient experience during surgery, particularly in larger and more controlled studies.

## Author contributions

**Conceptualization:** Murat Bayram Kaya, Arzu Esen Tekeli, Nurettin Kurt, Ali Kendal Oğuz.

**Data curation:** Arzu Esen Tekeli.

**Formal analysis:** Arzu Esen Tekeli.

**Methodology:** Arzu Esen Tekeli.

**Supervision:** Arzu Esen Tekeli, Nurettin Kurt, Mehmet Emin Keskin.

**Validation:** Arzu Esen Tekeli.

**Writing – original draft:** Murat Bayram Kaya, Arzu Esen Tekeli, Ali Kendal Oğuz.

**Writing – review & editing:** Arzu Esen Tekeli.
